# Hemocompatibility of cellulose phosphate aerogel membranes with potential use in bone tissue engineering

**DOI:** 10.3389/fbioe.2023.1152577

**Published:** 2023-04-19

**Authors:** Christian B. Schimper, Paul Pachschwöll, Manfred F. Maitz, Carsten Werner, Thomas Rosenau, Falk Liebner

**Affiliations:** ^1^ Department of Chemistry, Institute of Chemistry of Renewable Resources, University of Natural Resources and Life Sciences Vienna, Vienna, Austria; ^2^ Leibniz Institute of Polymer Research, Max Bergmann Center of Biomaterials, Dresden, Germany

**Keywords:** cellulose aerogel, cellulose phosphate aerogel, bioactivity, tetrabutylammonium fluoride, cell scaffolding materials, hemocompatibility

## Abstract

Cellulose is an appealing material for tissue engineering. In an attempt to overcome some obstacles with cellulose II cell scaffolding materials related to insufficient biomineralization, lack of micron-size porosity, and deficiency in surface charge, respective solutions have been proposed. These included covalent phosphorylation of different cellulose materials targeting relatively low degrees of substitution (DS 0.18–0.23) and processing these cellulose derivatives into scaffolding materials by a dissolution/coagulation approach employing the hitherto rarely used TBAF/DMSO/H_2_O system for cellulose dissolution. Here, we report bioactivity and preliminary hemocompatibility testing of dual-porous cellulose phosphate aerogels (contrasted with the phosphate-free reference) obtained via coagulation (water/ethanol), solvent exchange and scCO_2_ drying. Deposition of hydroxyapatite from simulated body fluid (7 days of immersion) revealed good bioactivity (1.5–2.2 mg Ca^2+^ per mg scaffold). Incubation of the scCO_2_-dried and rehydrated scaffolding materials in heparin anticoagulated human whole blood was conducted to study selected parameters of hemostasis (prothrombin F1+2 fragment, PF4, count of thrombocyte-leukocyte conjugates) and inflammatory response (C5a fragment, leukocyte activation marker CD11b). Adhesion of leukocytes on the surface of the incubated substrates was assessed by scanning electron and fluorescence microscopy (DAPI staining). The results suggest that phosphorylation at low DS does not increase platelet activation. However, a significant increase in platelet activation and thrombin formation was observed after a certain fraction of the negative surface charges had been compensated by Ca^2+^ ions. The combination of both phosphorylation and calcification turned out to be a potent means for controlling the inflammatory response, which was close to baseline level for some of the studied samples.

## Introduction

Tissue engineering has emerged in the last few decades as a promising, rapidly developing field of regenerative therapy, such as for bone repair. Biological substitution of missing, injured or diseased bone is accomplished by *in vitro* generation of artificial tissue combining pluripotent, vital cells with growth factors on a suitable carrier, commonly referred to as cell scaffolding material.

“Scaffolds are the masterpiece of bone tissue engineering” (Ghassemi et al., 2018). Similar to bone substitute materials, cell scaffolds have to comply with many requirements to support cell attachment, proliferation, migration and osteogenic differentiation (Perry, 1999; Nandi et al., 2010). This includes biocompatibility, lack of toxicity and inflammatory response, biodegradability, adequate overall mechanical strength, appropriate micron- and nanosized open-porous architecture, sterilizability, as well as suitable surface chemistry for controlled release of bioactive compounds (Hutmacher, 2000; Brown et al., 2009; Porter et al., 2009).

A wide variety of scaffold materials has been tested, including synthetic and natural polymers, bioceramics, metallic foams and bioactive composite materials. All of them have pros and cons. While bioceramics are among the strongest promoters of osteoblast adhesion and proliferation, they are relatively brittle (Ducheyne and Qiu, 1999; Stevens et al., 2008). Metal foams are lightweight, strong, biocompatible and osteoconductive but feature poor osseointegration, are prone towards corrosion-induced inflammatory response and are not biodegradable (Kao and Scott, 2007; Stevens et al., 2008; Nandi et al., 2010). Bioactive composite materials unifying beneficial features of their constituents (Wang, 2003) are particularly promising, since key features, such as controlled resorption, can be tailored particularly well (Ciardelli et al., 2005).

Among the three classes of natural polymers used for scaffolds. i.e., proteins, polysaccharides and polynucleotides (Lee and Shin, 2007; Short et al., 2015), cellulose has recently emerged as particularly interesting source for implantable materials (Poustis et al., 1994) or bone regeneration (Dias et al., 2003). However, there are a few obstacles to overcome. Common routes of cellulose hydrogel preparation afford open-nanoporous gels deficient in micron-size pores (Pircher et al., 2016), which is not favorable for cell ingrowth, proliferation and osteogenic differentiation. Furthermore, cellulose hydrogels obtained by consecutive dissolution of cellulose, solution casting and antisolvent-mediated coagulation are deficient in surface charge, which adds to impeded biodegradation and molecular recognition, and is non-supportive to crystallization of cd-Hap (Wan et al., 2006; Wan et al., 2007).

Therefore, we report here the results of a study that evaluates the response of the human blood system to negatively charged cellulose phosphate aerogel membranes for bone tissue engineering. Even though hemocompatibility might be of lesser importance in bone repair at first glance, it is actually the most realistic negative means to analyze inflammatory response (Raghavendra et al., 2015). Native cellulose is highly inflammatory since it activates the complement cascade through its abundant hydroxyl groups (Mahiout et al., 1987). Negatively charged surface moieties, such as phosphate groups, are on the other hand known triggers of contact activation, blood platelet activation, and blood coagulation (Nigretto et al., 1989; Sperling et al., 2009). After surgical incisions, repair begins with platelet clot formation, activation of the coagulation cascade, and platelet degranulation with release of growth factors (Everts et al., 2006). Blood clotting and blood platelet activation are, therefore, desirable effects in bone surgery as they stop bleeding, and the release of growth factors from activated platelets promotes wound healing and osteoconduction.

Aiming at circumventing potential drawbacks associated with inhomogeneous growth and precipitation of calcium-deficient hydroxlyapatite crystallites (cd-Hap) inside the nanoporous membranes as well as insufficient biomineralization, cellulose was subjected to phosphorylation prior to processing into the open-porous matrices. This was motivated by a series of studies showing that covalent grafting of phosphate groups onto various biopolymeric substrates, such as poly(ethylene terephthalate) (Kato et al., 1996), chitin (Yokogawa et al., 1997), cotton linters (Mucalo et al., 1995), microcrystalline cellulose (Granja et al., 2001a), Ca^2+^-crosslinked nanocellulose (Basu et al., 2017), or bacterial cellulose (Wan et al., 2006; Torres et al., 2012; Mulinti et al., 2018) improves bioactivity, osteoconduction and biocompatibility. It also enhances the levels of bone matrix proteins (Fang et al., 2009; Liu et al., 2009), such as bone sialoprotein, osteopontin (Hunter and Goldberg, 1994) and osteocalcin (Komori, 2020). Cellulose phosphates are non-toxic towards cultured human osteoblasts and fibroblasts (Granja et al., 2001b) similar to cellulose/calcium phosphate hybrid materials (Salama, 2019) including bacterial cellulose derived materials (Torgbo and Sukyai, 2018). Covalent phosphorylation was preferred over cd-Hap precipitation approaches (Ma et al., 2010) to reach a more homogeneous and complete biomineralization. A relatively low degree of substitution (DS) was targeted since higher phosphate contents were found to impair attachment and proliferation of human mesenchymal stem cells (MSCs) (Maia et al., 2013), human bone marrow stromal cells (hBMSC) as well as expression of osteocalcin and type I collagen (Granja et al., 2006). Aiming at a compromise scenario between desired platelet activation, release of growth factors and low inflammatory response, the introduced phosphate groups were balanced to various extent by calcium counter ions.

In a concomitant attempt to further optimize the open macroporous cell wall architecture of dual-porous biocompatible cellulose scaffolds presented elsewhere (Pircher et al., 2015), the scaffolding materials of this study were prepared following a recently proposed cellulose dissolution/coagulation protocol employing the hitherto rarely used cellulose solvent system tetrabutyl ammoniumfluoride/dimethyl sulfoxide/water (TBAF/DMSO/H_2_O) (Pircher et al., 2016; Schimper et al., 2018). The thus obtained cellulose aerogels and cellulose phosphate aerogels (Schimper et al., 2020) were tested for bioactivity (cd-Hap formation in simulated body fluid) and incubated with heparin anticoagulated whole human blood. Both blood and scaffolds were studied with regard to key parameters of hemostasis and inflammatory response.

## Materials and methods

All chemicals used for cellulose modification and preparation of the respective aerogels were of highest grade available. Cotton linters (CL) and bleached hardwood pre-hydrolysis kraft pulp (hwPHK) were provided by COST E41 action partners. Key characteristics, such as weight-averaged molecular weight and carbonyl group contents, have been reported elsewhere (Roehrling et al., 2002; Schimper et al., 2018).

• CL: M_W_ 146.2 kg mol^-1^, 4.0 μmol g^-1^ C=O
• hwPHK: M_W_ 164.3 kg mol^-1^, 8.4 μmol g^-1^ C=O

Prior to dissolution or phosphorylation, all cellulosic source materials were activated by disintegration in water (solid-to-liquid ratio 1:400, w/w). The obtained slurry was freeze-dried and stored at +4°C until further processing.

### Synthesis of cellulose phosphate

Cellulose phosphorylation was conducted as recently detailed (Schimper et al., 2020). The amounts given in the following are sufficient for the preparation of 100 g cellulose phosphate. The highly viscous phosphorylation reagent was prepared from phosphoric acid (354 g, derivatization agent, ≥85%), phosphorus pentoxide (250 g, binding of released water) and triethyl phosphate (185 mL, solvent) in an argon atmosphere (48 h). Subsequent phosphorylation of cellulose comprised repeated disintegration of the respective cellulosic substrate in water (ca. 1:400, w/v) and consecutive submersion in ethanol and *n*-hexanol (ca. 1:80, w/v, 2 × 24 h for each solvent). About 50% of *n*-hexanol was then replaced by the phosphorylation reagent. After shaking (50°C, 72 h), the modified cellulose was removed from the reaction mixture and washed consecutively with hexanol, ethanol, and deionized water. The last step was repeated until the filtrates were free of inorganic phosphate (negative molybdenum blue reaction). The obtained phosphorylated celluloses were freeze-dried and stored in desiccators until further use. Analysis of P contents and calculation of DS_P_ (Equation 1) was accomplished according to DIN ISO 11885 after microwave-assisted pressure digestion in HNO_3_/H_2_O_2_ (Suflet et al., 2006; Schimper et al., 2020).

$$DS_{P}=\frac{m_{P}\;\left[g\right]∙162.14\;\left[g∙{mol}^{-1}\right]}{m_{cell}\left[g\right]∙30.97\;\left[g∙{mol}^{-1}\right]∙}$$ (1)

### Preparation of cellulose and cellulose phosphate hydrogels and aerogels

The cellulose solvent used in this study was a solution of 16.6 wt% TBAF and 0.95 wt% H_2_O in DMSO, prepared as described previously (Schimper et al., 2020). In brief, TBAF∙3 H_2_O (157.55 g) was dissolved in DMSO (342.45 g), which was followed by lowering the water content to about 1.5% using activated molecular sieve (4 Å). After filtration, the above optimum composition with regard to cellulose dissolution performance was adjusted by dilution with dry DMSO. Defined volumes of the pre-heated solution (60 °C) were used to dissolve portion-wise the respective celluloses and cellulose phosphates to form microscopically clear solutions with solid contents of 2.5%. These solutions were cast into cylindrical molds (Ø 45 mm × 2 mm). Cellulose coagulation was accomplished by immersing the molds in 96% ethanol. Exhaustive washing with deionized water afforded transparent hydrogels. Conversion to aerogels was accomplished by solvent exchange to ethanol and subsequent scCO_2_ drying (Schimper et al., 2020). Both volume (±0.1 mm³) and weight (±1 mg) of the materials were recorded prior to and after scCO_2_ drying.

### Bioactivity in simulated body fluid (SBF)

The bioactivity in SBF was tested as described previously (Kokubo and Takadama, 2006). Three small cylinders (Ø 25 mm) were punched out from each of the hydrogel samples (CL, CL-P, hwPHK and hwPHK-P). They were repeatedly washed with 20 mL portions of deionized water prior to immersion in 10 mL SBF solution (2.5 mM Ca^2+^, 36.5 °C) for 6 days. Every 2 days, the substrates were transferred to fresh SBF solution. After 7 days, the incubated materials were washed with deionized water (3 × 20 ml) to remove excess SBF and non-bonded calcium ions. Each of the small disks was then agitated in 1.6 mL of 0.1 M HCl for 2 hours to liberate Ca^2+^ from hydroxyl apatite (Hap) potentially formed. An aliquot of the supernatant was mixed with a known volume of 13% HNO_3_ and subjected to ICP-OES (inductively coupled plasma atomic emission spectroscopy) analysis. The remaining specimens were dried at 120 °C overnight to determine their dry weight, required for calculation of the total amount of Ca^2+^ precipitated per gram of sample (Equation 2).

$$\omega_{{Ca}^{2+}}\;\left(mg∙g^{-1}\right)=\frac{m_{{Ca}^{2+}}\;in\;supernatant\;\left[mg\right]∙1000}{m_{dry\;sample}\;\left[mg\right]}$$ (2)

### Scanning electron microscopy (SEM)

The surface and interior of selected gold-sputtered samples was studied using a Philips XL30 (SEM) or Quanta 250 field emission gun (FEG-SEM) instrument with energy-dispersive X-ray (EDAX) mapping (acceleration voltage 5 kV).

### Hemocompatibility studies

Hemocompatibility tests were conducted using cellulose (reference) and cellulose phosphate aerogels that had been stored under dry conditions and were assumed to be sterile due to the conducted sequence of solvent exchange and scCO_2_ drying (Soares et al., 2019). These materials were placed between glass plates and hydrated with bidistilled water for 1 hour until dimensional changes had ceased. Soft pressure was applied to even out irregularities before all samples were equilibrated in 0.9% NaCl for at least 4 h. For blood compatibility evaluation, duplicates of each type of hydrated test membrane were mounted on top and bottom of the sterilized incubation chambers described elsewhere (Streller et al., 2003), providing a total scaffold surface of 6.25 cm^2^. To study the impact of different levels of calcification, selected samples were pretreated by filling the respective incubation chambers first with 500 µL of sterile aqueous 0.9 wt% NaCl solution supplemented by different quantities of calcium chloride (Table 1). Those samples were mounted onto an overhead shaker and gently revolved at 4 °C overnight. All non-calcified materials were treated in the same way, however, without supplementing the NaCl solution with CaCl_2_. Calculation of the amounts of CaCl_2_ to provide Ca/P ratios of 0.5, 1 and 2 was based on the phosphorous contents of CL-P (DS 0.185) and hwPHK-P (DS 0.228, Table 1).

**TABLE 1:** Samples used in the hemocompatibility assay.

Label	Description	Calcification molar Ca/P	Ca^2+^ content
			µmol mg^-1^
CL	cotton linters aerogels (reference)	--	--
	ρ_B_ = 33.5 mg cm^-3^		
CL Ca II	CL aerogels, immersed in 10 mL	--	--
	38.86 mM CaCl_2_ for 12 hours		
CL-P	aerogels from phosphorylated cotton linters, 37.20 mg P g^-1^, DS = 0.185	--	--
CL-P Ca I	CL-P aerogels, calcified	0.5	2.00
CL-P Ca II	CL-P aerogels, calcified	1.0	3.98
CL-P Ca III	CL-P aerogels, calcified	2.0	8.01
hwPHK	aerogels from hardwood prehydrolysis kraft pulp (reference) ρ_B_ = 41.1 mg cm^-3^	--	--
hwPHK-P	aerogels from phosphorylated hwPHK, 45.85 mg P g^-1^, DS 0.228	--	--
hwPHK-P Ca I	hwPHK-P aerogels, calcified	0.5	2.47
hwPHK-P Ca II	hwPHK-P aerogels, calcified	1.0	4.92

### Incubation of cellulosic scaffolds in human whole blood

Blood compatibility of the cellulosic scaffolds was probed with freshly drawn and heparin (2 IU mL^−1^) anticoagulated whole blood of two healthy donors (male, 34 years; female, 40 years, ABO matched), who did not take any medication for at least 10 days. The blood donations from two donors were pooled and 500 µL aliquots were transferred into the above-described chambers for incubating the cellulosic scaffolds at 37 °C for 2 hours, maintaining slow and constant revolution in the absence of an air-interface. Thereafter, the blood was withdrawn from the chamber, mixed with the appropriate stabilizers for Enzyme-linked Immunosorbent Assay (ELISA) analysis of platelet factor 4 (PF4; Haemochrom Diagnostica GmbH, Essen, Germany), prothrombin F1+2 fragment (Siemens Healthcare Diagnostics, Germany) and complement C5a fragment (DRG Instruments GmbH, Marburg, Germany) and further processed according to the instructions of the manufacturers. Samples were frozen at −80 °C for further analysis. The conducted blood compatibility tests of anti-inflammatory and anticoagulant cellulosic membranes were approved by the Ethics Commission of the State Medical Council of Saxonia, Dresden, Germany (approval No. EK-BR-37/09–1).

### Blood analysis after incubation with cellulosic scaffolds

Besides PF4, F1+2 fragment and C5a fragment (*cf.* above), aliquots of the incubated blood were subjected to cell staining for flow cytometry. The target was to differentiate between granulocytes and monocytes by employing:

• specific bonding of Allophycocyanin (APC) conjugated anti-CD14 (Clone M5E2, BD/Pharmingen) to a glycosylphosphatidylinositol (GPI)-linked membrane glycoprotein (known as LPS receptor) which is predominantly expressed by monocytes,
• specific bonding of phycoerythrin-conjugated anti-CD11b (clone ICRF44, Biozol, Eching, Germany) to the integrin alpha-M/beta-2 which shows activation-dependent expression by these two types of phagocytic cells,
• specific staining with PerCP-Cy5.5 conjugated anti-CD41a (Clone HIP8, BD/Pharmingen, Heidelberg, Germany) to a platelet-specific integrin involved in platelet aggregation and platelet adhesion to the ECM (extracellular matrix).

All staining procedures were conducted at room temperature (ca. 30 min). Cell activation during this period was suppressed by addition of NaN_3_, targeting a final concentration of 0.1 wt%. Cells were fixed, and red blood cells were lysed in a lyse-no-wash protocol using FacsLyse™ (BD Biosciences) solution. Analysis was performed by flow cytometry using a FACSCalibur™ (BD Biosciences, Heidelberg, Germany) instrument. Granulocytes were identified by their characteristic appearance in the forward vs side scatter plot and monocytes additionally by CD14 positivity (surface marker for monocytes and macrophages). In general, cells were collected until 10,000 granulocytes were measured. Their integrin CD11b expression (regulating leukocyte adhesion and migration to mediate the inflammatory response) was quantified as relative fluorescent units. The median intensity of the CD11b signal of the granulocyte population and the rate of CD41a positive events in the granulocyte or monocyte population, respectively, were quantified. An initial blood sample was stained immediately and served as reference. Positive controls were obtained by incubation of blood samples with 100 EU mL^-1^ lipopolysaccharide for 2 h at 37 °C.

### Analysis of cellulosic scaffolds after incubation with blood

Samples for microscopic analyses were washed blood-free with phosphate-buffered saline (PBS) and fixed with 4% paraformaldehyde in PBS. The surface cell population was analyzed after DAPI staining by fluorescent microscopy, taking 10 microphotographs at random positions on the sample surface and using computer-assisted evaluation for determining the cell count. For SEM analysis, the cells were fixed with 2% glutaraldehyde in PBS for 20 min and exchanged with PBS. Subsequently, samples were solvent-exchanged to absolute ethanol in five steps and dried under supercritical conditions using a BAL-TEC CPD 030 critical point dryer. Leukocytes, erythrocytes and thrombocytes attached to the surface were inspected in high vacuum mode using an ESEM XL 30 FEG (FEI-Philips, Eindhoven, Netherlands). The hemocompatibility assessment was performed in two independent experiments, with all samples typically being analyzed three times.

## Results and discussion

*Dimensional changes of samples:* To ensure safe storage and prevention from microbial infestation, all cellulosic hydrogels prepared were converted into aerogels by consecutive solvent exchange and scCO_2_ drying. Since the obtained light-weight open-porous materials are potentially prone towards shrinkage, their dimensional changes along the process chain of washing, solvent exchange, scCO_2_ drying and rehydration were studied. The data of Table 2 confirm moderate in-plain contraction of the discs by about 10%–15% of their original diameters throughout coagulation and solvent exchange. This, however, occurred for the reference samples only, while the cellulose phosphate gels were virtually stable already at the low DS values (0.18–0.23) adjusted. This is supposedly caused by electrostatic repulsion of the negatively charged cellulose nanoparticles formed upon coagulation, similar to aerogels from oxidized, nanofibrillated cellulose (Liebner et al., 2012). During scCO_2_ drying and long-time storage at ambient conditions both reference and cellulose phosphate gels shrink to a similar extent. However, it is worth noting that rehydration with bidistilled H_2_O swelled the phosphorylated scaffolds almost back to their original size (Ø 45 mm). The maximal loss in diameter was ≤7.3% (CL-P). Hydration with sodium chloride prior to incubation of the samples with whole human blood slightly enhanced the contraction (≤9.4%, hwPHK-P), presumably due to incorporation of hydrophilic hydroxyl groups into the [Na(H_2_O)_n_]^+^ (n = 1–7) hydration sphere and formation of micro-solvated sodium ion clusters (Ohtaki and Radnai, 1993; Tahoon et al., 2019).

**TABLE 2:** Contraction of the disk-type cellulosic scaffolds (Ø 45 mm) were measured by a caliper throughout the different steps of cellulose coagulation and solvent exchange, scCO_2_ drying, storage in air, hydration with bidistilled H_2_O and equilibration in 0.9 % NaCl. Measurements were repeated until no further volume contraction occurred. Total volumetric shrinkage in the sum of all process steps, including scCO_2_ drying and bulk density of the aerogels obtained. (Mean ± standard deviation, n = 8).

	CL	CL-P	hwPHK reference	hwPHK-P
	Reference			
	% shrinkage in diameter			
coagulation/solvent exchange	14.5 ± 2.1	2.5 ± 1.6	10.6 ± 3.1	0.9 ± 0.9
scCO_2_ drying	22.6 ± 4.9	18.0 ± 6.0	22.1 ± 2.0	16.4 ± 2.8
ambient storage (30 days)	33.4 ± 3.6	21.5 ± 2.4	27.5 ± 3.4	21.5 ± 2.8
hydration (ddH_2_O)	28.3 ± 5.2	7.3 ± 2.8	29.2 ± 2.4	5.5 ± 4.5
hydration (0.9% NaCl)	27.3 ± 2.9	9.1 ± 2.9	28.3 ± 1.8	9.4 ± 4.5
	% total shrinkage in volume			
casting → after scCO_2_ drying	36.9 ± 12.4	37.6 ± 11.3	33.8 ± 8.4	36.2 ± 10.0
	bulk density of aerogels [mg cm^-3^]			
casting → after scCO_2_ drying	59.4 ± 10.4	42.7 ± 10.3	62.4 ± 7.0	41.5 ± 5.2

### Bioactivity assessment

Bioactivity of the cellulose phosphate scaffolds (compared with the counterparts from non-derivatized cellulose) was assessed by formation of hydroxyapatite after 7 days of incubation in SBF (Kokubo and Takadama, 2006). The latter was prepared according to a refined protocol proposed 2003 by the TC150 ISO committee for *in vitro* prediction of the *in vivo* bonelike apatite-forming ability of implant materials, which is an strong support for artificial materials to bind to living bone (Bohner and Lemaitre, 2009; Mu et al., 2023).

The phosphorylated cellulosic scaffolds clearly showed bioactivity in terms of *in vitro* apatite formation on their surfaces, while the phosphorous-free reference materials were inert in this regard. It is noteworthy that compared to the CL-P sample (25.2 ± 5.6 mg Ca^2+^ g^-1^), more than twice the amount of apatite (determined as deposited Ca^2+^) was formed on hwPHK-P (55.1 ± 5.8 mg Ca^2+^ g^-1^).

### Aerogel morphology

**FIGURE 1:**
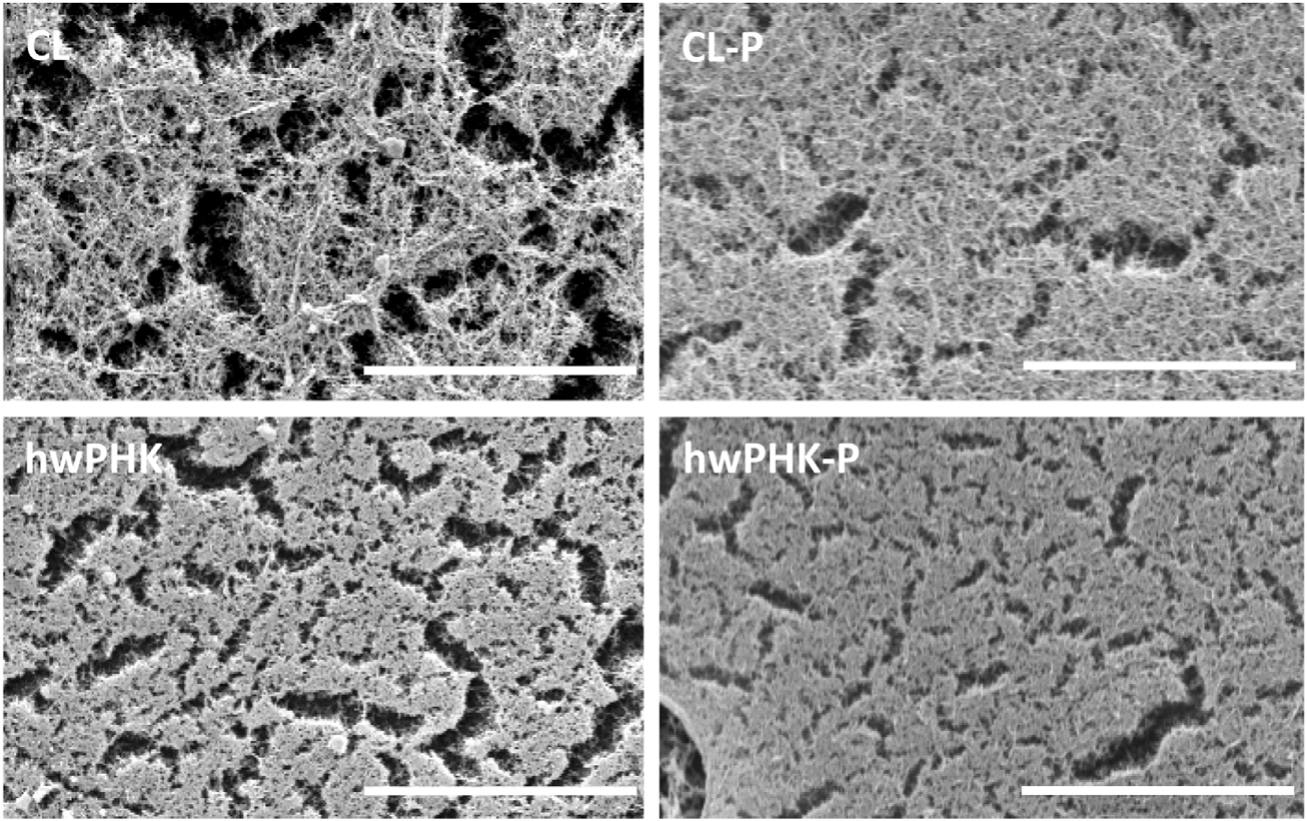
SEM photomicrographs of surface of reference and phosphorylated samples prior to hemocompatibility tests. Bar width: 5 µm.

The morphology of cellulosic networks formed during coagulation of cellulose from solution state—coinciding with an allomorphic cellulose I → II transition—largely depends on the solvent/antisolvent system used. This has been explained by different phase separation mechanisms (Sescousse et al., 2011; Hedlund et al., 2019) which can also affect the extent of cellulose II crystallinity, pore characteristics, size of the network forming entities, mechanical performance and even the chemical integrity of cellulose (Pircher et al., 2016). Here, we use TBAF/DMSO/H_2_O as a hitherto rarely used solvent system targeting scaffold materials. Advantages include quick cellulose dissolution even at high DP, far-reaching preservation of chemical integrity due to relatively mild processing conditions (60 °C), and formation of networks with interconnected micron-size pores. The scaffold structure is composed of an open-nanoporous substructure formed by small fibrils (Pircher et al., 2016; Schimper et al., 2018). SEM pictures in Figure 1 clearly show this dual-porous morphology for the scCO_2_-dried materials prepared from the non-phosphorylated cellulosic substrates. Microscopic evidence suggests that cellulose phosphates form somewhat denser networks at the expense of micron-size pores. Pore interconnectivity, pore volumes and pore size distributions, including the results of small-angle and wide-angle X-ray scattering (SAXS, WAXS) studies of similar samples have been reported elsewhere (Pircher et al., 2016; Schimper et al., 2018; Schimper et al., 2020). The reduced fraction of micron-sized pores is considered an advantage as it can impart enhanced robustness to the “walls” of large interconnected micron-size pores (50–500 µm) as present in dual-porous cellulosic scaffolds prepared from temporary templates of slightly fused PMMA or paraffin spheres (Pircher et al., 2015).

### Hemostasis parameters

Hemostasis is an important response to vascular injury, where the release of tissue factor and the expression of von Willebrand factor lead to blood coagulation and thrombocyte activation, adhesion and aggregation, causing blood to form a gel-like plug. The latter is capable of sealing a vessel and stopping bleeding (Aronson, 2016; Ruggeri, 2007). Biomaterials-induced coagulation, however, is commonly triggered by contact phase activation. This includes conformational changes and activation of factor FXII at negatively charged surfaces, reciprocal activation of Kallikrein (which also participates in inflammation) and activation of factor XI. In a secondary phase of hemostasis, a series of coagulation factors mediate the formation of thrombin, the key enzyme of the coagulation cascade. It is responsible for the actual blood clotting since it converts water-soluble fibrinogen into fibrin monomers, which eventually aggregate and get crosslinked to the insoluble fibrin clot. Thrombin is a strong activator of blood platelets, which, *vice versa*, accelerate the coagulation cascade.

A well-functioning hemostatic response is essential for tissue integrated implants or cell scaffolding materials to prevent blood loss at implantation. Further, growth factors, such as the Platelet Derived Growth Factor (PDGF), released from activated blood platelets, support wound healing and osseointegration. While chemokines and many growth factors provide a density gradient, triggering the migration of osteogenic cells, fibrin and structural proteins guide the movement of the cells towards the implant surface. The clinical application of platelet-rich plasma makes use of these mechanisms in an amplified way (Schlegel et al., 2004).

Hemocompatibility testing, comprising evaluation of thrombosis, coagulation, platelet function, hematology, and complement activation (Amoako and Gbyli, 2018), is one of the main tasks to evaluate non-endogenous foreign materials, designed to have long-term, intimate contact with the blood circuit (e.g., stents, heart valves, vascular prostheses). While these tests are frequently conducted with blood plasma, the use of whole blood is more informative since the extent of inflammatory and coagulant response depends on the complex interplay of cellular and humoral activations.

Direct analysis of thrombin for rating the total coagulation activation is limited by rapid inactivation. Therefore, indirect methods have been established, including the quantitative analysis of the prothrombin F1+2 fragment, which is an activation product that is cleaved from prothrombin upon conversion to thrombin by prothrombinase (clotting factor Xa). Alternatively, the stable thrombin-antithrombin (TAT) complex is quantified, which is formed in a fast reaction with the thrombin inhibitor antithrombin III in the presence of heparin. Release of the F1+2 fragment and the TAT complex are equivalent integral parameters of the thrombin formation, and they are formed at the same molar concentration.

**FIGURE 2:**
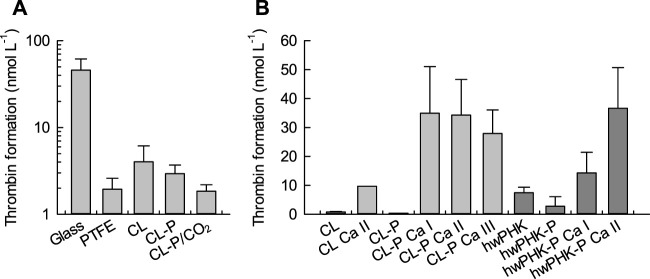
Cumulative thrombin activation as marker for plasmatic coagulation activation upon incubation of the cellulosic scaffolds and reference materials with whole human blood. **(A)**: Pre-study, DS_P_ 0.20, values calculated from Thrombin-antithrombin (TAT) complex concentration. **(B)**: Phosphorylated and calcified cellulosic materials; data obtained from prothrombin F1+2 fragment release.

Here, we compare hemostasis and inflammatory response for the above two types of cellulose phosphate aerogels and their phosphate-free counterparts after incubation with freshly drawn heparin-anticoagulated human whole blood. In a first screening (Figure 2A), the coagulation activity of cotton linters (CL) cellulose, its phosphorylated counterpart (CL-P) and rehydrated aerogels prepared from phosphorylated cellulose (CL-P/CO_2_) showed a relatively low coagulation activity of the phosphate-free cellulose II membranes, in the range of the inert, negative control PTFE and substantially lower than the positive control glass. This was even more pronounced for unprocessed phosphorylated cellulose (CL-P). Processing of CL-P into aerogels (CL-P/CO_2_) eventually afforded materials of very low hemostatic activation (Figure 2A).

In a subsequent set of samples, these results were confirmed by incubation of selected cellulose and cellulose phosphate gels with whole human blood (Figure 2B). Both non-phosphorylated cellulosic materials induced similarly weak thrombin activation. Since the negative surface charge of the aerogel membranes was anticipated to cause coagulation activation by the intrinsic pathway (Gorbet and Sefton, 2004; Miyamoto et al., 1990), the introduced phosphate groups were counterbalanced to different extents by calcium ions, targeting molar calcium-to-phosphate (Ca/P) ratios of 0.5, 1.0 and 2.0 (*cf.* Table 1). The data of Figure 2, however, show a minor effect of cellulose phosphorylation only, probably due to the low degrees of substitution. Partial calcification, on the contrary, strongly boosted coagulation. Positively charged surfaces can promote coagulation by binding Factor VII Activating Protease (FSAP (Sperling et al., 2017)), neutralizing heparin, or through adhesion of negatively charged blood platelets. The calcium dependence of most coagulation factors may contribute to the enhanced coagulation at the calcified cellulose samples. In this context, even the enhanced thrombin activation observed for the calcium-loaded aerogels from non-phosphorylated CL is understandable (CL/Ca II). It can be assumed that despite extensive washing after calcification, small quantities of calcium ions remained in the nanoporous gels physically immobilized either as part of the cellulose hydration spheres or as counter ion of carboxyl groups (Cl: 8.8 μmol g^-1^; (Liebner et al., 2012)). Assuming that this had happened to a similar extent for the other samples, baseline correction still yields a distinct activating effect on thrombin formation and coagulation for the calcified cellulose phosphate aerogels, in particular at a molar Ca/P ratio of 1.0.

**FIGURE 3:**
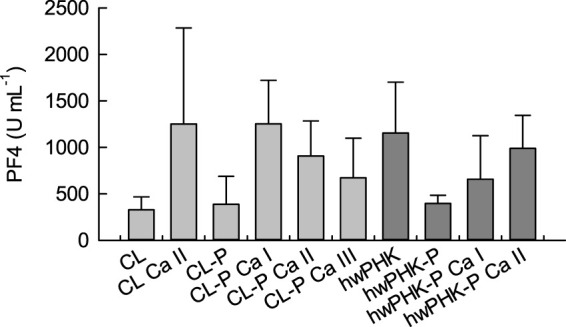
Platelet factor PF4 levels in whole human blood after 2 hours of incubation with the cellulose samples.

Figure 3 shows the concentrations of platelet factor 4 (PF4) in human whole blood after incubation with the different cellulosic samples. PF4 is a sensitive marker of thrombocyte activation and degranulation, since this cytokine peptide is stored in the α-granules of blood platelets together with growth factors, proteases and other cytokines, from where it is released upon degranulation after platelet activation (Pepper and Prowse, 1992). Physiologically, PF4 binds and neutralizes heparin, promotes blood coagulation, inhibits local antithrombin activity, exerts chemotactic attraction towards granulocytes (neutrophils) and fibroblasts, and appears to have anti-angiogenic effects (Aronson et al., 1977).

In agreement with the amounts of the thrombin activation (Figure 2), incubation of blood in contact with aerogels from both non-phosphorylated and phosphorylated cellulose resulted in slightly enhanced PF4 levels (Figure 3). Calcification significantly increased the PF4 release, corresponding to the distinctly elevated thrombin levels. Since this effect was observed to a similar extent for samples CL Ca II and CL-P Ca I, overcompensation of the relatively few negative charges (phosphate, carboxylate groups) and fixation of excessive calcium ions by the coordination spheres of cellulose in the aqueous environment (*cf.* above) is likely. If this was the case, the resulting positively charged surfaces might have contributed to platelet activation and release of PF4 (Miyamoto et al., 1990; Sagnella and Mai-Ngam, 2005). However, thrombocyte activation tended to decrease with increasing molar Ca/P ratio, which was suggested (less prominent) also in the plasmatic coagulation analysis (Figure 2).

**FIGURE 4:**
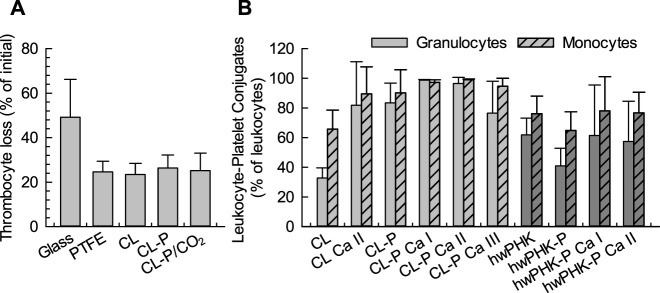
**(A)**: Thrombocyte loss during whole blood incubation with cotton linter materials DS_P_ 0.20. **(B)**: Amount of leukocyte-thrombocyte conjugates determined as CD41a positive leukocytes in flow cytometry after incubation of the respective cotton linters materials with whole human blood.

Platelet activation during incubation with foreign materials commonly leads to adhesion of the platelets to surfaces, to other cells, and to a fibrin clot (if formed), lowering the platelet count in the blood. Experiments with non-phosphorylated and phosphorylated cotton linters (samples CL and CL-P in Figure 4A) as well as with rehydrated aerogels prepared from phosphorylated cellulose (CL-P/CO_2_) suggested a moderate thrombocyte loss for all cellulosic materials of about 20%, comparable to that observed for PTFE. This can be attributed to the formation of leukocyte-thrombocyte conjugates, which was studied by flow cytometry, interpreting the appearance of the thrombocyte-specific surface marker CD41a on granulocyte and monocyte typical cells as conjugates with platelets (Figure 4B). The formation of leukocyte-thrombocyte conjugates is governed by binding of P-selectin of activated platelets to the P-selectin glycoprotein ligand-1 (PSGL-1) on leukocytes. Since P-selectin is expressed during platelet activation, platelet-leukocyte conjugates can be regarded as another marker of blood platelet activation (Arman et al., 2015) and can complement the above results of PF4 analysis.

Compared to the native cellulose reference materials CL and hwPHK, Figure 4B suggest slightly enhanced leukocyte-platelet conjugate formation for phosphorylated and an even more pronounced one for calcified samples, with enhanced participation of monocytes. The observed conjugate formation was somewhat more pronounced for the samples from cotton linters than from pre-hydrolysis kraft pulp (hwPHK). The latter has approximately the double amount of carbonyl groups (8.1 vs 4.4 μmol g^-1^ C=O) and a broader distribution of molar weight (Schimper et al., 2018), which is an indication for the presence of impurities that may lead to these small differences. However, virtually 100% monocyte and granulocyte conjugation was observed for both types of cellulose phosphates at a molar Ca/P level of 1.

### Inflammatory response

Immunological reactions towards foreign materials are governed by non-specific, lymphocyte-independent reactions. Complement activation on the alternative pathway, granulocyte and monocyte activation by complement fragments, by release products of blood platelets or directly by lipopolysaccharides are the most prominent reactions (Gorbet et al., 2019).

**FIGURE 5:**
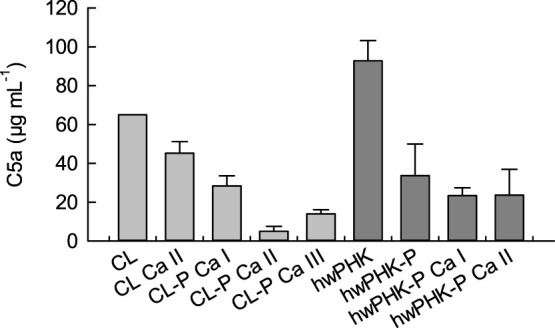
Complement C5a peptide after two hours of incubation of the cellulose samples with human whole blood.

Complement activation on the alternative pathway is initiated by fluid-phase C3-convertase mediated hydrolysis of C3 proteins and covalent binding of the released C3b fragment via its Gln991 residue to hydroxyl or amino groups of the surface of foreign materials (Liszewski and Atkinson, 1993; Sperling et al., 2007). C3b, together with other factors, forms the C5-convertase of the alternative pathway, which promotes the cleavage of C5 proteins into the highly inflammatory C5a peptide and the C5b fragment. C5b plays a central role, since its sequential binding to C6, C7, C8, and finally to C9 is essential for the formation of the membrane attack complex of the complement system. The plasma concentration of the soluble C5a fragment after incubation with the cellulosic substrates was, therefore, used as an indicator of complement activation (Figure 5).

The elevated levels of C5a found for both unmodified cellulosic samples are not surprising since cellulose is known to be highly complement activating due to its abundance of hydroxyl groups, which covalently bind the complement fragment C3b, thus activating the alternative pathway (see above; (Sperling et al., 2007)). However, treatment of the non-phosphorylated cotton linters gels with an aqueous solution of calcium chloride followed by exhaustive washing reduced the release of C5a already distinctly. This was even more pronounced for calcification of both cellulose phosphate gels. At a molar Ca/P ratio of one, the C5a levels dropped to less than 10% (CL-P) and 15% (hwPHK-P) of the C5a concentration determined for the unmodified reference samples, which is quite surprising considering the low DS values of both cellulose phosphates studied.

**FIGURE 6:**
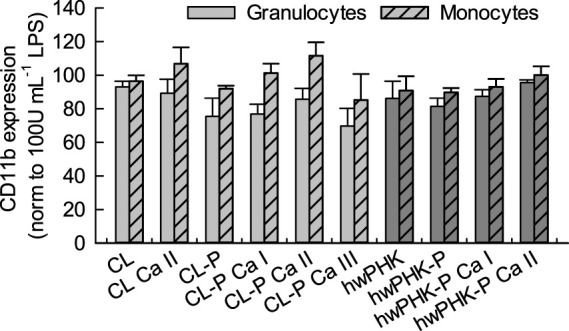
Leukocyte activation after incubation with cellulosic materials quantified by the expression of CD11b on leukocytes after incubation with whole blood.

Since the C5a peptide is one of the strongest activators of myeloid leukocytes, its concentrations (Figure 5) were compared with the expression of the leukocyte integrin (adhesion molecule) CD11b on the surface of granulocytes and monocytes, which serves here as a sensitive marker for leukocyte activation. The results shown in Figure 6 confirm a strong activation of granulocytes for the non-phosphorylated samples, which is in good agreement with the elevated release of C5a. Conjugation with phosphate groups, however, entailed a less pronounced reduction of the inflammatory potential than expected.

**FIGURE 7:**
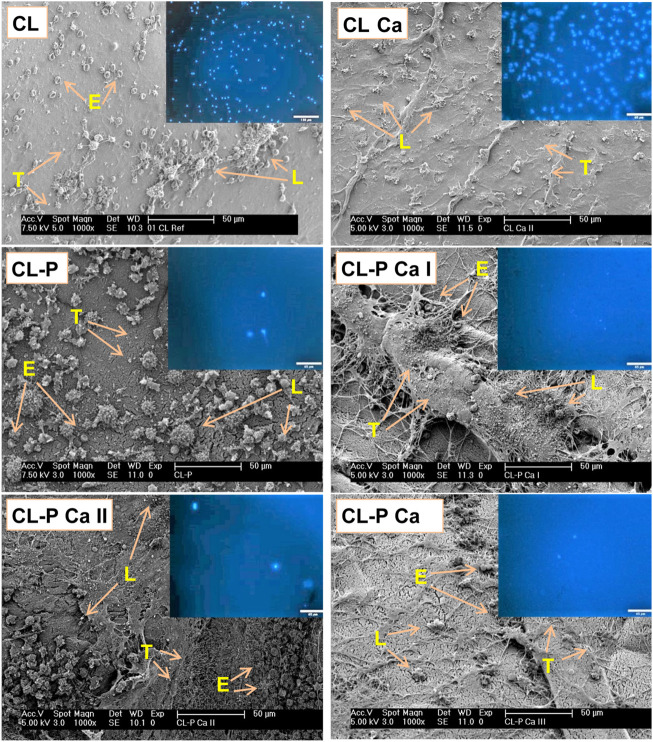
SEM and fluorescence images (inserts) of the (aero)gel membranes of the cotton linters sample series after incubation with whole human blood (E: erythrocytes, L: lymphocytes, T: thrombocytes). Bright blue dots in the fluorescence images represent the nuclei of adherent leukocytes (DAPI staining).

Blood analysis was complemented by (aero)gel surface analysis using SEM and fluorescence microscopy to visualize and count deposited thrombocytes and leukocytes. The SEM micrographs of Figure 7 show an inhomogeneous cell distribution on the surface of the samples. All adherent cells - erythrocytes (E), leukocytes (L) and thrombocytes (T) - were found in relatively high number on the surface of the reference samples. Red blood cells with their characteristic biconcave shape weakly attached to the surface; leukocytes were widely spread under the formation of many pseudopodia, and platelets were found to be adherent. Phosphorylation alone induced almost fibrin-free surfaces with adherent monocytes (bigger) and granulocytes (smaller). Phosphorylation and calcification apparently reduced the cell count. Calcification induced the most pronounced formation of fibrin among the phosphorylated materials. The number of leukocytes distributed within the inhomogeneous fibrin networks of the phosphorylated and calcified samples was clearly decreased. The absence of pseudopods suggested that the adhering leukocytes were not highly active. Platelets adhered to cellulose surfaces and to the crossings of the fibrin network.

**FIGURE 8:**
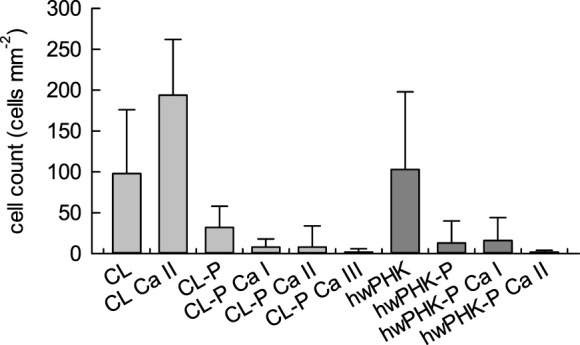
Cell count of granulocytes and monocytes counted on random places of fluorescent images (n=10).

For counting leukocytes on the surfaces of the incubated (aero)gel membranes, the cells were permeabilized, and their DNA was stained using the membrane impermeable fluorescence dye 4′,6-diamidino-2-phenylindole (DAPI) to facilitate observation by fluorescence microscopy. Among the three leukocyte populations, i.e., lymphocytes, monocytes and granulocytes, which are the only nucleated cells in blood, only monocytes and granulocytes tend to adhere to surfaces. Therefore, the bright-blue stained cell nuclei visible in the fluorescence micrograph insets of Figure 7 (CL sample series) mainly represent the granulocyte and monocyte populations. The cell counts of these phagocytic cells are shown in Figure 8. The data confirm the pronounced recognition of the non-derivatized cellulosic matrices as foreign materials, further increasing after calcification. This observation correlates well with the clearly enhanced activation of platelets (*cf.* PF4 release of Figure 3), which boosts the recruitment of leukocytes. Phosphorylation, at the low degrees of substitution investigated here, surprisingly suppressed recruitment and adhesion of leukocytes. This was even more pronounced for some of the calcified samples, depending on both type of cellulose and level of calcification. There, virtually leukocyte-free (aero)gel membrane surfaces were obtained, suggesting a significantly reduced complement activation and C3b binding. These data were in good agreement with the levels of both C5a and CD11b determined in plasma after incubation (*cf.* Figure 5).

## Conclusion

This study investigated two types of cellulose II aerogels from phosphorylated cotton linters and hardwood prehydrolysis kraft pulp with regard to hemocompatibility in terms of hemostasis and inflammatory response. The cellulosic surfaces moderately activate plasmatic coagulation (but to a clearly lesser extent than known from glass) and trigger a moderate inflammatory response via the alternative pathway of the complement cascade, leading to granulocyte and monocyte activation and adhesion on incubated surfaces. Even at the low degrees of substitution, phosphorylation reduces plasmatic coagulation (thrombin formation). Moreover, slightly reduced leukocyte activation (CD11b) was seen for the phosphorylated cotton linters and the pre-hydrolysis kraft pulp cellulosic substrates. Partial calcification and compensation of the negative surface charges turned out to be a potent means of activating blood platelets for degranulation, beneficial for osteoconduction and osseointegration. In addition, calcification suppressed inflammatory complement response and leukocyte activation almost entirely. These promising results encourage us to extend the studies to other types of cellulose-based cell scaffolding materials that can offer an even more appropriate dual porosity. Respective materials, featuring interconnected micron-size pores (50–500 µm) embedded in networks of open-nanoporous cellulose (phosphate) struts (Pircher et al., 2015), are expected to provide a better environment for MSC migration and ingrowth, for example.

## Data Availability

The original contributions presented in the study are included in the article, further inquiries can be directed to the corresponding author.
